# The Role of Scientific Research in Human Papillomavirus Vaccine Discussions on Twitter: Social Network Analysis

**DOI:** 10.2196/50551

**Published:** 2024-05-09

**Authors:** Geneviève Jessiman-Perreault, Jean-Christophe Boucher, So Youn Kim, Nicole Frenette, Abbas Badami, Henry M Smith, Lisa K Allen Scott

**Affiliations:** 1 Alberta Health Services Calgary, AB Canada; 2 School of Public Policy University of Calgary Calgary, AB Canada; 3 Department of Oncology University of Calgary Calgary, AB Canada; 4 Department of Community Health Sciences University of Calgary Calgary, AB Canada

**Keywords:** human papillomavirus, HPV, vaccine, immunization, social media, misinformation, social network analysis

## Abstract

**Background:**

Attitudes toward the human papillomavirus (HPV) vaccine and accuracy of information shared about this topic in web-based settings vary widely. As real-time, global exposure to web-based discourse about HPV immunization shapes the attitudes of people toward vaccination, the spread of misinformation and misrepresentation of scientific knowledge contribute to vaccine hesitancy.

**Objective:**

In this study, we aimed to better understand the type and quality of scientific research shared on Twitter (recently rebranded as *X*) by vaccine-hesitant and vaccine-confident communities.

**Methods:**

To analyze the use of scientific research on social media, we collected tweets and retweets using a list of keywords associated with HPV and HPV vaccines using the Academic Research Product Track application programming interface from January 2019 to May 2021. From this data set, we identified tweets referring to or sharing scientific literature through a Boolean search for any tweets with embedded links, hashtags, or keywords associated with scientific papers. First, we used social network analysis to build a retweet or reply network to identify the clusters of users belonging to either the vaccine-confident or vaccine-hesitant communities. Second, we thematically assessed all shared papers based on typology of evidence. Finally, we compared the quality of research evidence and bibliometrics between the shared papers in the vaccine-confident and vaccine-hesitant communities.

**Results:**

We extracted 250 unique scientific papers (including peer-reviewed papers, preprints, and gray literature) from approximately 1 million English-language tweets. Social network maps were generated for the vaccine-confident and vaccine-hesitant communities sharing scientific research on Twitter. Vaccine-hesitant communities share fewer scientific papers; yet, these are more broadly disseminated despite being published in less prestigious journals compared to those shared by the vaccine-confident community.

**Conclusions:**

Vaccine-hesitant communities have adopted communication tools traditionally wielded by health promotion communities. Vaccine-confident communities would benefit from a more cohesive communication strategy to communicate their messages more widely and effectively.

## Introduction

### Background

Cervical cancer is one of the most preventable types of cancer in the world. Almost all cases are attributable to human papillomavirus (HPV), for which an effective vaccine exists [1]. Part of the global strategy to eliminate cervical cancer includes fully vaccinating 90% of girls with the HPV vaccine by the age of 15 years [2]. However, the global HPV immunization coverage currently remains suboptimal [3]. While many countries are experiencing vaccine supply issues, even high-income countries with reliable vaccine supply and comprehensive school-based programs are still failing to meet vaccine targets, largely due to vaccine hesitancy [4].

Studies show that people now search the web for health information more often than they talk to health professionals about these matters [5]. The popularity of social media platforms has also created a phenomenon wherein people not only use the web to access health information but also play an active role in cocreating the information and ideas (in the form of opinions, anecdotes, and links to other sources of information) that they encounter in these web-based spaces [6]. Social media spaces create an important setting for people to interact and for communities to emerge, as they are not geographically bound but rather reflect patterns of shared interests, purpose, or identities [7]. As such, vaccine-confident and vaccine-hesitant groups represent distinctive ideologies and create distinctive web-based communities. The distinction between these 2 groups lies in their attitudes, beliefs, and behaviors associated with vaccine decision-making, in that vaccine-confident groups reflect public trust in vaccines and the evidence supporting their efficacy, effectiveness, and safety, which leads to their uptake of recommended vaccines. Vaccine-hesitant groups, for their part, tend to doubt this information, demonstrated by their reluctance or refusal to receive recommended vaccines [8,9].

Despite a large body of evidence demonstrating the safety and efficacy of the HPV vaccine [10,11], attitudes toward the vaccine and the accuracy of information shared about this topic in web-based settings vary markedly from extremely negative and erroneous to supportive and factually accurate [12]. In addition, in recent years, there has been a rapid increase in the accessibility of scientific journals and subsequent dissemination of scientific findings through social media [13]. Simultaneously, there has been a decline in the role of unbiased science journalists and other communication experts as mediators between scientists and the public [14]. While these changes have had a democratizing effect on scientific knowledge and allowed for better communication between scientific communities and the public, this unfiltered access to scientific research also creates an environment where individuals may have difficulty in differentiating valid and credible information from biased and unreliable information or may misinterpret legitimate findings [15]. In contrast, researchers have also noted that the growth of open science can create opportunities for people to discuss novel research across polarized boundaries [16], but the type and quality of scientific research about HPV vaccination that is being shared in web-based discussions is unknown. Finally, with a wealth of open-access scientific research available, there are concerns about how ideologically motivated communities, such as vaccine-hesitant groups, integrate scientific knowledge into their social media communication strategies to amplify uncertainty around vaccines [17]. It is prudent to investigate how scientific research is integrated into web-based HPV vaccine discussions, given that web-based information is typically considered to be more credible, reliable, and authoritative if supported by scientific citation, notwithstanding the source of journal, authorship, or other features [18].

Twitter (recently rebranded as *X*; as data collection occurred before the rebrand, we will be using its former name throughout this paper) is one of the largest, most popular, and most influential social media platforms in the world. Twitter has also traditionally been a preferred source of public opinion data for applied public health research [19-22]. This is because social media feeds such as Twitter offer an avenue for continuous, near-real–time collection of unsolicited information generated by many individuals regarding a variety of topics of interest [23,24]. Several studies have recently demonstrated the benefits of leveraging social media over traditional methods such as surveys as a source of primary data for health promotion interventions, including those aimed at increased participation in HPV immunization programs [25].

### Objectives

Exposure to web-based discussions about HPV immunization on Twitter, regardless of geographic location, may influence peoples’ attitudes toward the vaccine [22,26,27]. Thus, there is significant interest among public health professionals to better understand how scientific knowledge about HPV immunization is wielded on Twitter, both to understand the impact of scientific knowledge on vaccine hesitancy and to identify opportunities for novel interventions aimed at countering or debunking misinformation and supporting increased uptake of the HPV vaccine [6,28]. Therefore, in this study, we aimed to do the following:

1. Describe and visualize the vaccine-hesitant and vaccine-confident communities’ patterns of sharing HPV vaccination–related scientific literature on Twitter
2. Thematically analyze the scientific literature shared by both vaccine-hesitant and vaccine-confident communities using a typology of research evidence
3. Determine whether there are differences in shares, quality of evidence, and other bibliometric indicators of the scientific literature shared by each community

## Methods

### Overview

**Figure 1:**
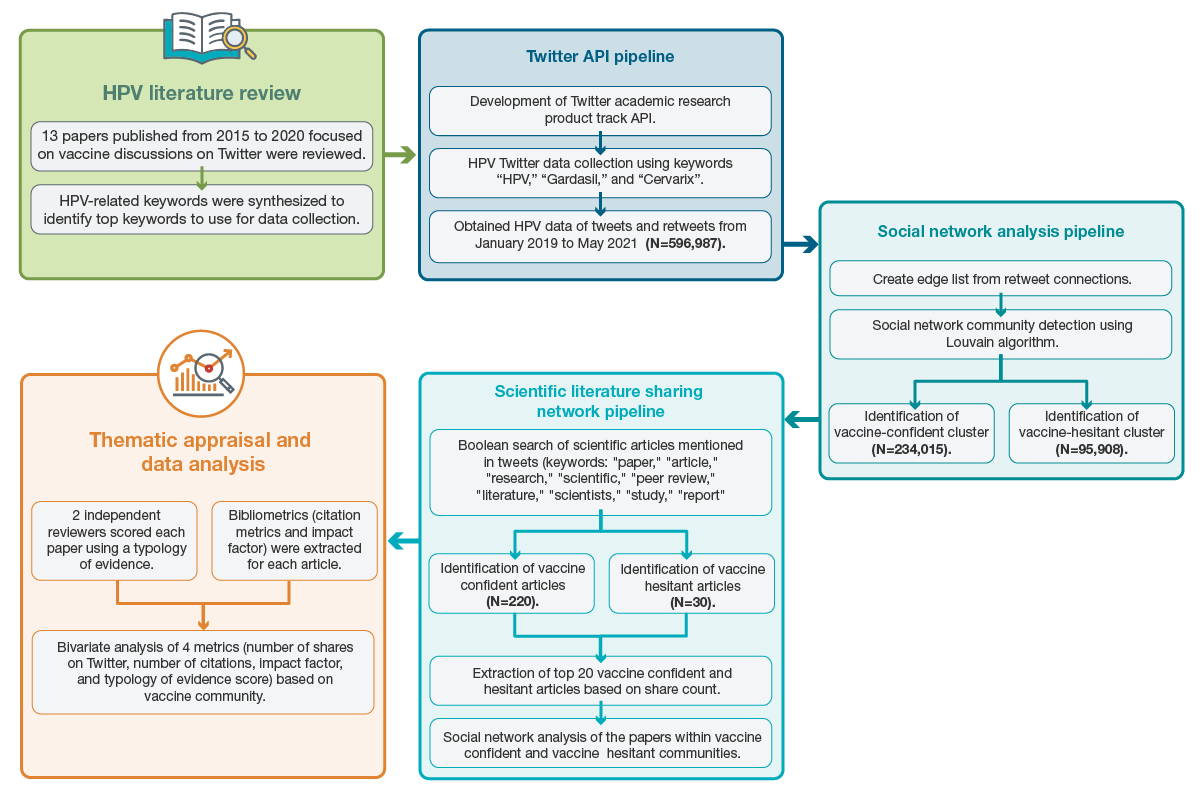
Summary of the study methods. API: application programming interface; HPV: human papillomavirus.

Our methods followed a multistep process. First, we conducted a rapid review to inform HPV and HPV vaccine keywords. Second, we used these keywords to filter tweets and create a data set. Third, we detected vaccine-confident and vaccine-hesitant communities and generated social network maps of each community based on tweets and retweet. Fourth, we detected the mentions of scientific literature in each community and extracted those papers for future statistical and social network analysis. A summary of these methods is presented in Figure 1 (adapted from the paper by Elyashar et al [29]), and further details are presented in the following sections.

### Literature Review to Inform Data Collection

To determine the most applicable keywords to guide this study, a rapid review was first conducted to determine the most frequently used keywords in literature focused on HPV and HPV immunization discourse on Twitter. The rapid review methodology was selected due to its efficiency in synthesizing a large volume of information in a timely yet systematic manner [30]. This review yielded 13 papers published between 2015 and 2020 about the topic of HPV immunization discussions on social media, with 11 (85%) focusing on HPV immunization discussions on Twitter specifically. We extracted the keywords used in each paper to filter content on social media (Textbox 1). Then, we synthesized these keywords to compile a list of the most used keywords to represent HPV and HPV vaccine discussions on social media, and the top 3 keywords were used to generate the data set.

**Papers yielded from the rapid review and the associated human papillomavirus (HPV)–related and HPV vaccine–related keywords.**

**Papers and keywords**

- Shapiro et al [31]
  “Gardasil,” “Cervarix,” “HPV AND vaccin*,” and “cervical AND vaccin*”
- Massey et al [32]
  “HPV,” “HPV vaccine,” “HPV shot,” “Gardasil,” and “Cervarix” (and hashtag equivalents)
- Keim-Malpass et al [33]
  “#HPV” and “#Gardasil”
- Du et al [21]
  “HPV,” “human papillomavirus,” “Gardasil,” and “Cervarix”
- Nelon et al [34]
  “#vaccines,” “#vaccine,” “#vaccinations,” and “#vaccination”
- Surian et al [35]
  “HPV AND vaccine,” “HPV AND vaccination,” “Gardasil,” “cervical AND vaccination,” “cervical AND vaccine,” and “Cervarix”
- Zhou et al [36]
  “HPV,” “vaccine,” “Gardasil,” “Cervarix,” “vaccination,” “cervical,” and “cancer”
- Becker et al [37]
  “Pentavalent OR pentavac OR quinvaxem”
- Dyda et al [38]
  “Cervical,” “Cervarix,” “HPV,” “human papillomavirus,” “vaccine,” “vaccination,” and “Gardasil”
- Chakraborty et al [20]
  “HPV,” “papilloma,” “pappiloma,” “papiolma,” “papillomavirus,” “Gardasil,” “Gardisil,” “Guardisil,” “Guardasil,” “Cervarix,” “cervical shot,” “cervical shots,” “cervical vaccine,” “cervical vaccines,” “cervical vax,” “cervical vaxine,” “cervical vaxines,” “cervical vaxx,” “cervical vaxxine,” “cervical vaxxines,” “cervical vaccination,” and “cervical vaccinations”
- Dunn et al [39]
  “Gardasil,” “Cervarix,” “HPV AND vaccine,” and “cervical AND vaccin”
- Budenz et al [40]
  “HPV,” “HPV vaccine,” “HPV shot,” “Gardasi,” and “Cervarix” (and hashtag equivalents)
- Zhang et al [41]
  “Cervarix,” “Gardasil,” “HPV,” “human papillomavirus,” “Gardasil,” “HPV AND vaccin*,” and “cervical AND vaccin*”

### Data Collection

Using 3 of the most common keywords that emerged from the initial rapid review (“HPV” OR “Gardasil” OR “Cervarix”), a data set of tweets and retweets was created (N=596,987). Then, tweets were collected using the Academic Research Product Track application programming interface (API) from January 2019 to May 2021 [42]. Data were collected using the Twitter API Python wrapper (Python Software Foundation, version 3.8.5) [43]. The construction of the API, data collection, and data processing (ie, importing, exporting, and filtering of data) were performed in Python [44].

### Ethical Considerations

This study received an exemption from ethics approval as determined by The Conjoint Faculties Research Ethics Board at the University of Calgary. This was due to its use of only publicly available information from an existing data set. Furthermore, the published results have omitted all identifiable information and are only presented in aggregate form.

### Social Network Analysis

First, we created a social network of accounts by creating an edge list using retweets. The retweet edge list consisted of nodes representing individual Twitter accounts and edges representing accounts that are being retweeted. The individual Twitter accounts were identified using the “username” information from the API, and the source of the retweet account information was extracted using the account mentions beside the “RT” in the tweets’ text in our data set. Our data set consisted of 57,109 retweets and 25,898 original or quoted tweets. Retweet networks were analyzed as they are found on aggregate to better reflect agreement among users and thus represent an ideological community on issues such as vaccination [45]. Second, we used a Louvain modularity method to classify subclusters of web-based communities in the resulting social network [46]. This method was chosen because the algorithm was designed to accurately detect subcommunities within large networks and operate fast computationally. Third, the social network analysis map also illustrated a strong polarization of the subclusters. Through this polarization and the identification of primary influencers within a subcommunity, the vaccine-confident (n=234,015) and vaccine-hesitant (n=95,908) web-based communities were identified. The primary influencers were detected by measuring the degree centrality, which is the measure of the number of connections each user has within the network. Thus, the accounts with the highest measure of degree centrality were categorized as primary influencers, as a high degree centrality demonstrates a high number of connections an account has within the network. These primary influencers, along with the content of the account’s bio descriptions and tweets, were qualitatively studied to examine their expressed positions regarding HPV vaccination. Edge list was constructed using Python, and the retweet social network analysis was conducted using Gephi- (Gelphi, version 0.9.2) [47].

### Scientific Literature Sharing Network Analysis

From the vaccine-confident and vaccine-hesitant data sets, we identified tweets that either mentioned or shared scientific literature through a Boolean search for tweets with an embedded http secure link or any of the select list of words (“paper,” “article,” “research,” “scientific,” “peer review,” “literature,” “scientists,” “study,” and “report”) [48]. This filter identified 220 papers from the vaccine-confident community and 30 papers from the vaccine-hesitant community. The titles of or links to these papers were extracted from the data set along with associated metrics such as number of shares for further analysis (as described in the *Data Analysis* section). We identified the top 20 most shared scientific publications in these respective communities. We chose to identify the top 20 most shared scientific publications due to the proportion of shares that these papers had—accounting for >97% of shares in the vaccine-hesitant community and approximately 61% in the vaccine-confident community. Then, we repeated the social network analysis steps by creating a retweet network of accounts sharing the top 20 prominent scientific publications within the vaccine-confident and vaccine-hesitant communities. The edge list for the vaccine-confident community comprised 989 nodes and 1013 edges, whereas the vaccine-hesitant group had 355 nodes and 422 edges. The primary influencers in this network were again identified using degree centrality measures, and we qualitatively analyzed these accounts on Twitter through their Twitter bio descriptions. The social network analysis of the scientific papers was conducted using Gephi (version 0.9.2) [47].

### Typology of Evidence for Thematic and Critical Appraisal

Overall, 2 members of the research team (GJP and NF) with subject area expertise in HPV immunization independently reviewed all scientific papers from each network using a typology of evidence, proposed by Gray [49], based on the suitability of the study design for the research question posed. This typology was determined to be the most appropriate and feasible approach to critically appraise the scientific papers because it allowed for the ability to schematically differentiate between diverse study designs (from in vivo to clinical trials and reviews). First, we classified the objective, research question, or aim of the study based on 9 categories that were used to classify research papers based on the typology by Gray [49] (presented in the first column of Table 1). Next, we classified each paper according to the study design. On the basis of these 2 metrics, a score ranging from 0 to 2 was assigned to each paper, where 0 indicates the least appropriate study design for the research question posed and 2 indicates the most appropriate design for the research question posed (refer to Table 1 for details about the scoring of the typology of evidence). The same 2 members of the research team compared their classifications and scoring, and if consensus could not be reached, a third member of the research team (LKAS) made the final decision. In addition, we extracted information about the characteristics of the paper (study design, research question, or objective), journal (journal name and year published), and author (names, affiliations, and conflicts of interest; refer to Multimedia Appendices 1 [50-70] and 2 [52,71-89] for results of the top 20 most shared papers obtained from the vaccine-confident and vaccine-hesitant communities). These data were used to conduct bibliometric analyses of the journal and descriptive analysis of the research content shared by each community, which are further described in the following sections.

**Table 1:** A typology of evidence (example questions in columns refer to human papillomavirus [HPV] vaccination for the prevention of cancer) based on appropriateness of study design for the research question posed (adapted from the papers by Gray [49] and Petticrew and Roberts [90]).

	In vivo and in vitro studies	Qualitative research	Cross-sectional survey	Case-control studies	Cohort studies	RCTs^a^	Quasi-experimental studies	Nonexperimental evaluations	Scoping reviews and narrative reviews
Effectiveness (does this work? does doing this work better than doing that?)	0	0	0	0	1	2	1	0	2
Process of service delivery (how does it work?)	0	2	1	0	0	0	0	1	2
Salience (does it matter?)	0	2	2	0	0	0	0	0	2
Safety (will it do more harm than good?)	0	1	0	1	1	2	1	1	2
Acceptability (will the focus population be willing to or want to take up the HPV vaccine?)	0	2	1	0	0	1	1	1	2
Cost-effectiveness (is it worth delivering this service?)	0	0	0	0	0	2	0	0	2
Appropriateness (is this the right service for this population?)	0	2	2	0	0	0	0	0	1
Satisfaction with the service (is this population satisfied with the service?)	0	2	2	1	1	0	0	0	0
Basic science (what is the cellular mechanism of action?)	1	0	0	0	0	0	0	0	0

^a^RCT: randomized controlled trial.

### Bibliometric Indicators

Traditionally, the prestige and quality of a journal was evaluated using citation metrics such as impact factor [91]. In the past few years, as assessment of scientific information has grown exponentially, new tools have been developed to capture the visibility and reach of web-based scientific information. Examples of these alternative metrics or altmetrics include likes, shared tweets, and retweets [92]. To compare traditional scholarly measures of quality to altmetrics, we collected data about the number of times the paper was shared by each vaccine community and the impact factor of the journal the paper was published in. We also collected data about the number of citations each shared paper had received through Google Scholar. Given that citations are impacted by the length of time since publication, we used the SCImago Journal Ranking (SJR) indicator, which provides a weighted average score that remains consistent each year and accounts for the prestige of the citing journal and the differences across subject fields, allowing for more equal comparisons across subject fields [93]. Each paper was assigned an SJR indicator, where a lower score indicates lower-ranking journals and higher scores indicate higher-ranking journals [94]. Journals that were not indexed in the Scopus database were not assigned an SJR score and were marked as missing in our database. These metrics were used to assess the influence of the shared papers in scientific research and the prestige of the journal the shared papers were published in.

### Data Analysis

Once these bibliometrics and typology-of-evidence scores were collected in a data set, basic descriptive results of these 4 metrics (number of shares on Twitter, number of citations, impact factor, and typology of evidence score) were calculated using median and IQR, given their skewed distributions. We also performed the Mann-Whitney *U* test, given the nonnormal distribution of these data [95], to determine whether there were statistically significant differences in the 4 indicators between the papers shared in the vaccine-hesitant and vaccine-confident communities. The four indicators examined were (1) the number of shares that the original tweet sharing the publication on Twitter received, (2) the SJR score of the journal the paper was published in, (3) the number of citations the paper received, and (4) the typology of evidence score that the paper received. Statistical significance was determined using *P* value <.05. Effect size was calculated using Cohen *d*, where a standardized difference of 0.2 indicates a small difference, difference of 0.5 indicates a medium difference, and difference of 0.8 indicates a large difference [96]. All data analyses were conducted using SAS Studio (SAS Institute, version 3.6).

## Results

### Overview

In total, 250 scientific papers (n=30, 12% in the vaccine-hesitant community and n=220, 88% in the vaccine-confident community) shared between January 2019 and May 2021 were identified. These papers received a combined total of 2247 shares on Twitter, with 562 (25.01%) shares for vaccine-hesitant papers and 1685 (74.99%) shares for vaccine-confident papers. On average, vaccine-hesitant papers received approximately 19.2 (SD 35.6) shares, whereas vaccine-confident papers received approximately 7.7 (SD 30.5) shares. Of these 250 scientific papers, the top 20 most shared papers from each vaccine community were used to produce a social network map of all tweets interacting with or sharing scientific papers about the HPV vaccine on Twitter (Multimedia Appendix 3).

### Vaccine-Hesitant Social Network

**Figure 2:**
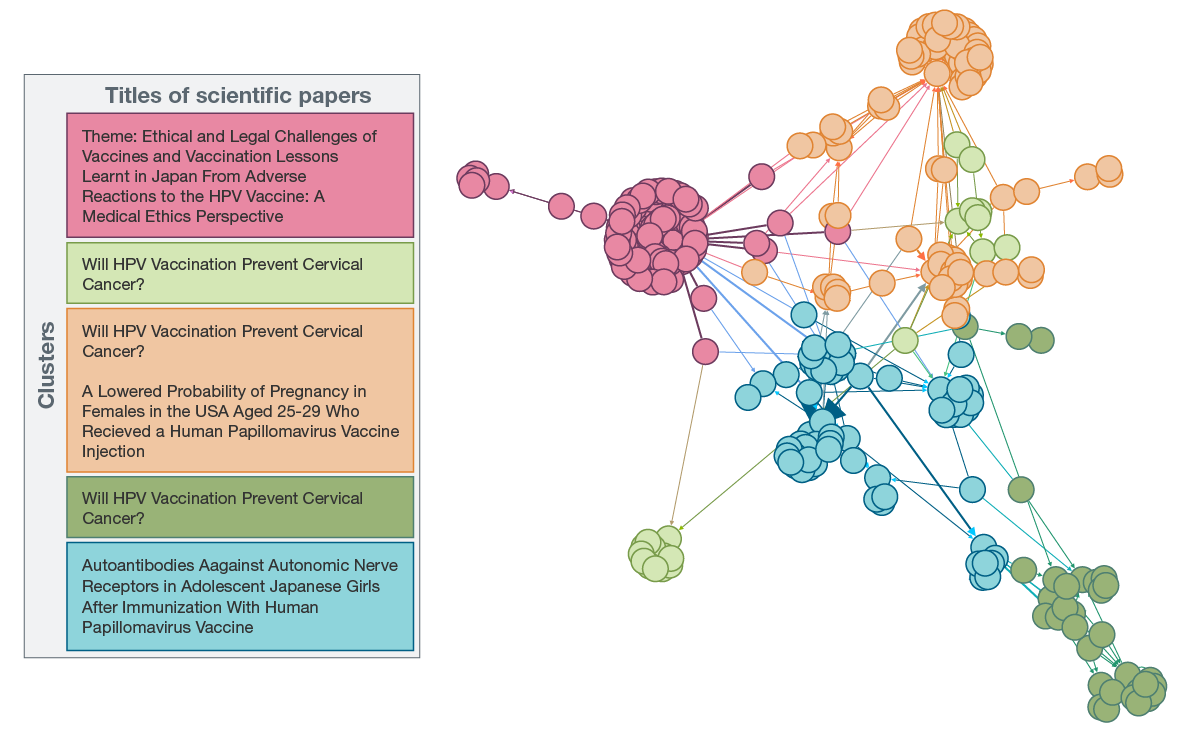
Network analysis of the vaccine-hesitant community sharing scientific research on Twitter. HPV: human papillomavirus.

Figure 2 presents the social network of all tweets sharing or interacting with tweets discussing scientific papers among the vaccine-hesitant community. As can be seen in Figure 2, the retweet network of scientific literature in the vaccine-hesitant community can be categorized into 5 distinct subclusters. Accounts associated with the red cluster shared papers focusing on the safety and ethical considerations around vaccination, with a journalist from a conservative news network emerging as the most influential account holder in this cluster. The most commonly shared paper in this cluster was a case study about the safety of the HPV vaccine in the context of alleged adverse reactions to the HPV vaccine in Japan [50]. In the light green cluster, 1 particular influencer, whose account was later suspended by Twitter, was similarly influential by sharing a paper focused on the effectiveness of HPV vaccination in the prevention of cervical cancer, namely, a widely circulated review paper about this topic [51]. Leading accounts linked to the orange cluster and the dark green cluster were personal user accounts, and both shared the same paper as the light green cluster, calling into question the efficacy of the HPV vaccine in the prevention of cervical cancer.

The orange cluster of the vaccine-hesitant community circulated a retracted paper, which alleged that HPV vaccines affected the vaccine recipients’ fertility and focused on safety [52]. Furthermore, the orange cluster’s location in the network (ie, adjacent to the light green cluster) suggests social influence and connection between the 2 clusters. In contrast, there was little interaction between the accounts in the light green cluster and the dark green cluster, suggesting that the influential accounts in these clusters independently found the same scientific literature and circulated it among a relatively isolated cohort of users. Finally, in the blue cluster, a European support group for those who had experienced vaccine injuries was the leading influential account, whereas a medical society’s account that published a widely shared paper in this cluster [51] was an account of secondary influence. Again, the influential accounts in this cluster shared scientific papers, which were retweeted by accounts that are more peripheral to the central clusters of influential accounts. The primary scientific paper circulated among users in this cluster focused on the theme of safety of the HPV vaccine by measuring the serum levels of autoantibodies in a cohort of girls who had possible adverse reactions following the receipt of the HPV vaccine [53].

### Vaccine-Confident Social Network

**Figure 3:**
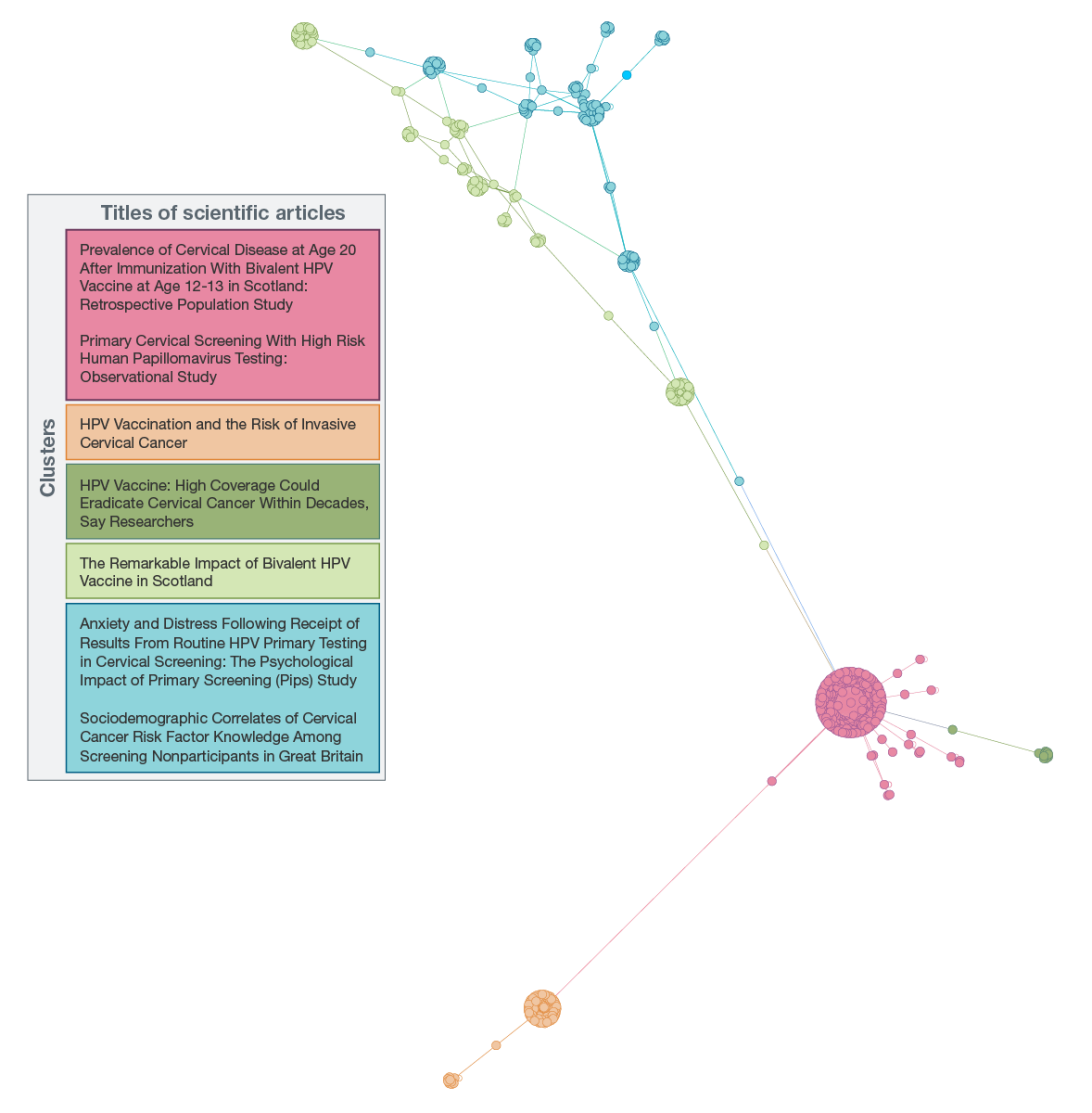
Network analysis of the vaccine-confident community sharing scientific research on Twitter. HPV: human papillomavirus.

The retweet network of scientific research shared among the web-based vaccine-confident community can similarly be divided into 5 distinct subclusters, as shown in Figure 3. The red cluster primarily included users retweeting literature from the *British Medical Journal*. There were 2 main papers circulated in this cluster, both of which focused on the effectiveness of the HPV vaccine. The first was a retrospective population study about the efficacy of the HPV vaccine in the prevention of cervical cancer in Scotland, focusing on the theme of satisfaction with service [71], whereas the second was an observational study about the outcomes of HPV screening in high-risk populations in England [72]. In the orange cluster, we observed a similar influence exerted by a government-funded public health agency, which shared a popular paper about effectiveness, focusing on the potential of the HPV vaccine to lower the risk of cervical cancer in a cohort population [73].

In the red, orange, and dark green clusters, there were physicians and health care workers among the users who retweeted influential tweets. For example, in the orange cluster, 1 particularly influential physician circulated an editorial paper about the effectiveness of the HPV vaccine, which indicated that high HPV vaccine coverage could eradicate cervical cancer within a few decades [74]. A science correspondent for a pre-eminent American newspaper was the leading influencer in the light green cluster wherein the primary paper circulated was an editorial, also focused on effectiveness, related to the positive impacts of HPV vaccination in Scotland [75]. Finally, in the blue cluster, a leading cancer prevention researcher from a British research institute was the leading influencer and author of the scientific papers circulated. In this cluster, papers about the psychological impacts of HPV screening [76] and the sociodemographic correlates of cervical cancer risk among those who did not participant in cervical screening programs in the United Kingdom [77] were recirculated by the accounts influenced by the leading researcher. Unlike the other clusters, health care workers were not overrepresented in the light green and blue clusters.

Overall, results from the vaccine-confident community suggest that health care, scientific, and news media communities are operating in closed systems. As we can see in Figure 3, there are relatively few bridging connections among the different communities discussing influential HPV vaccination literature in the vaccine-confident space. In contrast, the vaccine-hesitant space (Figure 2) is a more cohesive and tightly connected community, suggesting that there are stronger knowledge flows between subclusters in this group. Twitter accounts in the vaccine-hesitant community appear to be more efficient in sharing information than the more fragmented vaccine-confident community (Multimedia Appendix 3). Furthermore, the vaccine-hesitant Twitter accounts are more effective in communicating the results and research of interest to one another, whereas those in the vaccine-confident space appear to struggle to disseminate the research of interest beyond their personal and professional communities. These findings are supported by the descriptive statistics presented later in the paper, which indicated that while the vaccine-confident community shares far more scientific papers than the vaccine-hesitant community, the scientific literature shared by the vaccine-confident community received far fewer shares per paper despite being published in higher-ranked journals.

### Typology of Evidence and Bibliometric Analysis

Table 2 presents the distribution of typology of evidence categorized by vaccine community type. Most of the scientific papers shared by the vaccine-hesitant community focused on safety (16/30, 55%) or effectiveness (8/30, 28%), exemplifying the key concerns legitimizing vaccine hesitancy. The vaccine-confident community shared papers related to a wider range of research themes, the most common being papers that focused on basic science (56/220, 25.7%), effectiveness (55/220, 25.2%), acceptability (49/220, 22.5%), and salience (38/220, 17.4%). While the level of focus on effectiveness was similar between the 2 communities, there was very little overlap in the specific papers selected for sharing.

**Table 2:** Description of the typology of evidence of all papers shared on Twitter categorized based on vaccine-confident and vaccine-hesitant communities.

	Vaccine-confident community (N=220), n (%)	Vaccine-hesitant community (N=30), n (%)
Effectiveness	55 (25)	8 (26.7)
Safety	12 (5.5)	16 (53.3)
Process of service delivery	4 (1.8)	1 (3.3)
Satisfaction with the service	1 (0.5)	0 (0)
Salience	38 (17.3)	1 (3.3)
Acceptability	49 (22.3)	1 (3.3)
Cost-effectiveness	5 (2.3)	0 (0)
Basic science	56 (25.5)	3 (10)

Table 3 presents the descriptive statistics about the 4 metrics for all the scientific papers shared by vaccine-confident (220/250, 88% papers) and vaccine-hesitant (30/250, 12% papers) communities. The 4 metrics described in Table 3 are the median shares per paper, the median number of citations each shared paper received, the median SJR score of the journal that published each shared paper, and the median typology of evidence score. Table 3 also presents the results from the Mann-Whitney *U* test. Tweets containing scientific papers shared by the vaccine-confident community received a median of 3 shares, compared to a median of 4 shares by the vaccine-hesitant community. Results from the Mann-Whitney *U* test indicate that there are statistically significant differences (*P*=.01) in shares of tweets containing papers about HPV vaccination between the vaccine-hesitant and vaccine-confident communities and that this difference is small (Cohen *d*=0.37). Scientific papers shared by the vaccine-confident community received a median of 13 citations compared to a median of 17 citations for the scientific papers shared by the vaccine-hesitant community. We did not find evidence of statistically significant differences in the number of citations received by papers shared between the vaccine-confident and vaccine-hesitant communities. Scientific papers shared by the vaccine-confident community received a median SJR score of 1.83 compared to a median score of 0.84 for the papers shared by the vaccine-hesitant community. Results from the effect size calculation found this to be a medium standardized difference (Cohen *d*=61). The Mann-Whitney *U* test also found evidence of statistically significant (*P*<.001) differences in SJR scores of the HPV-related papers shared between the vaccine-confident and vaccine-hesitant communities. Finally, scientific papers shared by both the vaccine-confident and the vaccine-hesitant communities received a median typology of evidence score of 1, and results from the Mann-Whitney *U* test did not find evidence of a statistically significant difference.

**Table 3:** Results from the Mann-Whitney U test for shares, number of citations, SCImago Journal Ranking (SJR), and typology of evidence score categorized based on human papillomavirus vaccine-confident and vaccine-hesitant communities.

	Vaccine-confident community (n=220), median (IQR)	Vaccine-hesitant community (n=30), median (median)	*P* value	Effect size (Cohen *d*)
Shares	3 (1.0-6.5)	4 (2.0-15.0)	.007	0.37
Number of citations	13 (5.0-75.0)	17 (9.0-44)	.28	0.19
SJR	1.83 (1.25-3.44)	0.84 (0.68-1.30)	<.001	0.61
Typology of evidence	1 (0.0-10)	1 (1.0-1.0)	.22	0.14

## Discussion

### Principal Findings

The increase in the volume of scientific publications shared on the web [13] and the growth of open-access scientific publishing [16] have created an environment of greater access to scientific literature among lay audiences. However, little is known about how scientific literature is being incorporated into web-based communication strategies of vaccine-confident and vaccine-hesitant communities. Our study examined how scientific literature focusing on the HPV vaccine is being shared by vaccine-hesitant and vaccine-confident networks on Twitter. We found that despite the increased quantity of scientific literature being shared, such literature is often used by the vaccine-hesitant community to proliferate misinformation about vaccination, which is amplified in a web-based environment such as Twitter. Therefore, Kata [97] has described four key tactics that are used by the antivaccination movement to spread their messages on the web: (1) skewing the science, (2) shifting the hypotheses, (3) censorship, and (4) attacking the critics. A study conducted by van Schalkwyk et al [17] demonstrated that vaccine-hesitant groups are strategic in their use of scientific literature on social media to amplify uncertainty about vaccine safety and that vaccine-hesitant accounts who use large arsenals of scientific literature play important roles in dissemination of information across multiple communication networks. Findings from our thematic analysis of the papers shared by the vaccine-hesitant networks confirm this. Our study also found that the vaccine-hesitant community was much more likely to share scientific publications that questioned the safety and effectiveness of the HPV vaccine, whereas the vaccine-confident community shared scientific publications on a wider range of topics. This aligns with the tactic of skewing the science (identified by Kata [97]), which focuses on criticizing scientific studies while simultaneously calling for more studies, particularly focusing on the need for randomized controlled trials that compare vaccinated children and unvaccinated children. Moreover, most of the papers shared by the vaccine-confident community focused on basic science (ie, in vitro or in vivo studies), and this focus lowered the typology of evidence score of the vaccine-confident community, while failing to contribute to a unified message in the vaccine-confident community.

Furthermore, the quality of journals that published the papers shared in these communities varied markedly. The scientific publications shared by the vaccine-confident community were significantly more likely to be published in higher-ranked journals and therefore obtained higher SJR scores, compared with those shared by the vaccine-hesitant community. Other researchers have found that critical appraisal is often absent when vaccine-hesitant individuals share “scientific evidence” on the web, which often includes citations that blur the line between legitimate scientific publications and fraudulent studies [98]. However, there is little evidence of communication across networks, despite repeated calls from public health communication experts to prebunk and debunk vaccine misinformation on the web [99,100]. Notably, both communities share a retracted paper, but their framing of the paper varies. The vaccine-confident community mocks the paper for its outlandish claims, whereas the vaccine-hesitant community highlights the findings as if they were accurate. This highlights 2 issues. First, despite not supporting the findings of the retracted paper, the vaccine-confident community still shared the paper, thus amplifying its reach. Second, the vaccine-hesitant communities’ definition of “scientific evidence” does not align with accepted norms, as retracted papers can no longer be considered part of the scientific evidence base.

Vaccine-hesitant groups have been shown to co-opt the perceived authority of professional sources (eg, WebMD and the American Medical Association) to bolster their claims, even when the associated evidence does not support their arguments [101]. Interestingly, past studies have shown that while both groups point out knowledge deficits in their counterparts and attempt to correct misinformation by offering alternate sources of evidence, vaccine-confident groups have been shown to infrequently cite scientific evidence to correct misinformation or present counterarguments in web-based forums [102]. However, our analysis shows that the vaccine-confident community often shares scientific literature on the web as a form of self-promotion or knowledge translation, rather than as a tool to counter misinformation or correct misinterpretations.

Consequently, consistent with others in this field, we suggest that vaccine researchers should take a more active role in the HPV-related conversations that are occurring on the web, beyond simply promoting their own studies and instead countering misinformation and disinformation on the web [103]. Researchers and practitioners hoping to meaningfully contribute to the conversation about HPV vaccination on the web should explore training in science communication and social media engagement strategies, including the monitoring and correcting of public misinterpretation of their studies on various social media platforms [103,104]. Studies show that the way in which health information is communicated affects recipients’ perception of it, with transparent communication fostering trust in health authorities and reducing the proliferation of conspiratorial beliefs [105].

### Limitations

While Twitter provides us with a large body of unfiltered discussions to examine, the use of Twitter is not universal, and younger individuals (aged 18-29 years) and minority groups tend to be overrepresented on Twitter [20,24]. Therefore, while this analysis is not universal for all demographics, such as those who do not use Twitter as a social media platform, it provides opportunities to collect information about the health opinions held by members of several priority populations. While this study provides a way of studying web-based social interaction, further studies are needed to understand vaccine hesitancy among the general population who may not use Twitter.

The creation of the data set of HPV-related and HPV vaccine–related tweets was based on 3 commonly used hashtags derived from a rapid review of published papers; therefore, there is the potential that we missed some tweets that also discussed HPV and HPV vaccine but were not captured by these hashtags. In addition, we extracted a variety of metrics about the papers and journals included in our data set, but given the wide variation in study design among the extracted papers, conducting a formal critical appraisal of quality was unfeasible for this project and is an area for future study. Furthermore, this study did not measure the engagement rate of tweets, which is a new analytic metric offered by Twitter and is calculated by dividing the number of engagements (ie, total number of times a user interacted with a post including retweets, replies, likes, and follows) by the number of impressions (ie, number of times a user is shown a particular post in their timeline or search results). It should be reinforced that the number of shares of a tweet is not equivalent to the impact of the content shared.

Another limitation is that one of the metrics collected in our study was the number of citations each paper had received, for which we chose to use the “cited by” count provided by Google Scholar. While there has been criticism about the *cited by* metrics provided by Google Scholar due to double counting of citations from published journals and other sources [51], Google Scholar covers a larger breadth of sources (eg, conference papers and book chapters) than alternative platforms such as Web of Science [106]. Finally, the time frame we selected to collect tweets for this study, that is, January 2019 to May 2021, presents a limitation. We chose to expand our data collection to 2021 to allow us to acquire a sufficiently large data set, because the COVID-19 pandemic began shortly after the start of our data collection period. With the emergence of the COVID-19 pandemic, health discussions on Twitter became heavily focused on COVID-19 instead of other topics, including HPV vaccination. We ultimately extended our data collection time frame beyond our original timeline to provide us with a sufficiently large corpus of tweets to analyze. Given the unique period of data collection (ie, before and during the COVID-19 pandemic), which influenced the quantity of discussion about non–COVID-19 topics, the generalizability of these findings is reduced. Our experience in collecting these data over the course of the COVID-19 pandemic has been explored further in another publication, where we examined the attitudes and sentiment on Twitter toward HPV vaccination amidst the context of the pandemic [107].

### Strengths

This study contributes to the growing body of knowledge about the discussions about HPV immunization in web-based settings by using novel mixed methods to identify what papers about HPV and HPV vaccine are being shared on the web and how vaccine-confident and vaccine-hesitant communities are using this knowledge in their web-based communication strategies. Our study demonstrates that vaccine-hesitant communities are using strategies of scientific authority by presenting them as “scientific evidence” on Twitter, regardless of the quality of the papers themselves. Vaccine-confident communities do not appear to be sharing papers to build consensus, rather they share their scientific studies. These findings are relevant to health communication experts who aim to combat vaccine misinformation and disinformation on the web by providing them with concrete examples of papers used to create distrust in HPV vaccines. Moreover, HPV researchers and health promotion organizations that use Twitter might find these results helpful in crafting a more deliberative knowledge translation strategy.

Our study has several strengths. First, we used a large body of data from Twitter to track near–real-time conversations about HPV vaccination on the web. Twitter, in its previous iteration, was one of the largest and most popular social media platforms and was seen as a preferred source of public opinion data for applied public health research due to the following features: (1) quick processes for collecting data sets, (2) low costs for data collection, (3) ability to monitor trends over time, and (4) ability to avoid researcher biases that are inherent to the design and delivery of traditional research tools such as surveys [21,24]. Therefore, this data set provided us access to a large number of unfiltered discussions from populations that are traditionally difficult to access through conventional data collection methods.

Next, our use of social network analysis allowed us to examine how scientific literature is shared and its connection within wider networks representing communities of interest. Thus, we were also able to identify key influencers within networks who potentially act as leverage points to amplify future health communication campaigns, while also shedding light on the density of vaccine-hesitant influencers compared to vaccine-confident influencers within the respective social networks. Finally, while the vaccine-hesitant community has attempted to use or distort scientific literature to support their viewpoints for a long time, to the best of our knowledge, this is the first study to examine how scientific evidence has been used and shared on the web by comparing both vaccine-hesitant and vaccine-confident web-based communities in discussions specifically related to the HPV vaccine.

### Conclusions

Many of the communication strategies initially used by health promotion communities, including the use of the logical fallacy such as appealing to scientific authority and scientific knowledge, appear to have been co-opted by the vaccine-hesitant community and are being used to create controversy by focusing on questions about the effectiveness and safety of the HPV vaccine. While the scientific literature shared within these vaccine-hesitant communities is often published in lower-ranked journals, they deliver a substantially more successful, coordinated strategy when it comes to communicating about HPV vaccine on Twitter, compared to the vaccine-confident communities. By widely sharing a curated selection of scientific publications among like-minded individuals, the vaccine-hesitant community members’ communication around the HPV vaccine yields much more interaction (ie, shares and retweets) than is observed in the vaccine-confident community’s efforts to disseminate research findings. While the scientific literature shared by members of the vaccine-confident community is published in higher-ranked journals, these papers receive far fewer interactions and have lesser reach on Twitter.

While the vaccine-hesitant community has successfully incorporated communication tools that were traditionally wielded by health promotion communities to advance their agenda, the web-based vaccine-confident community could benefit from paying attention to their dissemination techniques for using web-based platforms such as Twitter to amplify their messaging. However, it is crucial that the vaccine-confident community’s messages ultimately be transmitted in a manner that fosters long-term trust and credibility, which stems from accurate and transparent communication.

## Appendix

Multimedia Appendix 1 Summary of the top 20 most shared scientific papers on Twitter by the vaccine-hesitant community.

Multimedia Appendix 2 Summary of the top 20 most shared scientific papers on Twitter by the vaccine-confident community.

Multimedia Appendix 3 Retweet network map of human papillomavirus immunization conversations (N=596,987).

## Abbreviations

API: application programming interface
HPV: human papillomavirus
SJR: SCImago Journal Ranking
