# Supplementary material for: The Role of Scientific Research in Human Papillomavirus Vaccine Discussions on Twitter: Social Network Analysis
Source: JMIR Infodemiology. 2024 May 9;4:e50551. doi: 10.2196/50551 (PMC11117132; doi:10.2196/50551)
Supplement: Multimedia Appendix 1 [file infodemiology_v4i1e50551_app1.docx]

Supplementary A: Summary of Top 20 Most Shared Scientific Articles on Twitter by Vaccine Hesitant Community

| Article Name | Authors and Year | Publication Name | Study Design | Conflict of Interest: Financial | Conflict of Interest: Personal | Research Objective | Shares on Twitter |
| --- | --- | --- | --- | --- | --- | --- | --- |
| Will HPV vaccination prevent cervical cancer? | Rees, Brhlikova & Pollock, 2020 | Journal of the Royal Society of Medicine | Scoping review, narrative reviews, rapid reviews | None stated | None stated | To describe the uncertainties generated by the design of Phase 2 and 3 efficacy trials for prevention of cervical cancer and its precursors and how they affect the interpretation of efficacy data. | 156 |
| Lessons learnt in Japan from adverse reactions to the HPV vaccine: a medical ethics perspective | Beppu, et al., 2017 | Indian Journal of Medical Ethics | Non-experimental evaluations (case study) | None stated | All the authors are members of Medwatcher Japan. Two of the authors are lawyers for the plaintiffs in the HPV vaccination lawsuits. | To review the adverse reactions following human papilloma virus (HPV) vaccination in Japan, and the measures taken by the Ministry of Health, Labour and Welfare (MHLW) to withdraw active recommendation of the vaccine. | 102 |
| Autoantibodies against autonomic nerve receptors in adolescent Japanese girls after immunization with human papillomavirus vaccine | Hineno et al., 2019 | Annals of Arthritis and Clinical Rheumatology | Cohort studies | None stated | None stated | To investigate autoantibodies against diverse protein coupled receptors in the serum of girls who complained of possible adverse effects after HPV vaccination | 79 |
| Comparison of HPV prevalence between HPV-vaccinated and non-vaccinated young adult women (20–26 years) | Guo, Hirth, & Berenson,2015 | Human Vaccines & Immunotherapeutics | Cross sectional survey | None stated | None stated | To compare type-specific HPV prevalence between vaccinated and unvaccinated young adult women using data from NHANES 2007–2012 | 52 |
| RETRACTED ARTICLE: [A lowered probability of pregnancy in females in the USA aged 25–29 who received a human papillomavirus vaccine injection] | DeLong, 2018 | Journal of Toxicology and Environmental Health | Cohort studies | None stated | The author filed a claim under the Vaccine Injury Compensation Program on behalf of her daughter. The Special Master dismissed the claim due to untimely filing. The claim did not include the HPV vaccine. | To analyze information [birth outcomes] gathered in National Health and Nutrition Examination Survey, which represented 8 million 25-to-29-year-old women residing in the United States between 2007 and 2014 | 31 |
| Adolescent premature ovarian insufficiency following human papillomavirus vaccination | Little & Ward, 2014 | Journal of Investigative Medicine: High Impact Case Reports | Non-experimental evaluations (case study) | None stated | None stated | To describe the cases of three young women who developed premature ovarian insufficiency following quadrivalent human papillomavirus (HPV) vaccination presented to a general practitioner in rural New South Wales, Australia. | 22 |
| Brachial plexus neuritis following HPV vaccination | Debeer, De Munter, Bruyninckx, Devlieger, 2008 | Vaccine | Non-experimental evaluations (case study) | None stated | None stated | To present a 19-year-old girl who developed a left brachial plexus neuritis following vaccination with a quadrivalent human papillomavirus (HPV) vaccine. | 15 |
| Screening for human papillomavirus in a low- and middle-income country | Atkinson, Mandujano, Bejarano, Kennedy, & Tsongalis, 2019 | Journal of Global Oncology | Cohort studies | One author received funding from MSD Oncology, Kinex, Pfizer, Asofarma. One author received funding from Illumina, QuanDx, Pillar Biosciences, Biocartis. The same author also holds stock at Chromacode received honouraria from ChromaCode, Seracare, AccuGenomics. | None stated | To determine the frequency of hrHPV type among women working in a manufacturing facility in San Pedro Sula, Honduras. | 15 |
| Signal detection of human papillomavirus vaccines using the Korea Adverse Events Reporting System database, between 2005 and 2016 | Ran, Yang, Lee, Kim, Choi, & Shin, 2019 | International Journal of Clinical Pharmacy | Cohort studies | None stated | None stated | To analyze signals associated with HPV vaccines using the Korean spontaneous AEs reporting system and data-mining methods and compare the results to current vaccine label information in South Korea and the United States of America, United Kingdom, European Union, and Japan to detect signals not currently listed on the labels. | 13 |
| Benefits and harms of the human papillomavirus (HPV) vaccines: systematic review with meta-analyses of trial data from clinical study reports | Jørgensen, Gøtzsche, & Jefferson, 2020 | Systematic Reviews | Systematic reviews and meta-analysis | One author has acted as a consultant for Roche (1997–1999), GSK (2001–2002), Sanofi-Synthelabo (2003) and IMS Health (2013). In 2014–2016, this author was a member of three advisory boards for Boehringer Ingelheim. They was a member of an independent data monitoring committee for a Sanofi Pasteur clinical trial on an influenza vaccine. | One author spoke by video link about the HPV vaccines at the International Federation for Injured Children and Adults conference in 2018 but received no fee or reimbursement for this.  Two of the authors were co-signatories of a complaint to the European Ombudsman on maladministration in relation to the EMA investigation of possible harms from HPV vaccines.  In 2011–2014, one author acted as an expert witness in a litigation case related to the antiviral oseltamivir, in two litigation cases on potential vaccine-related damage and in a labour case on influenza vaccines in healthcare workers in Canada. | To address the uncertainties of the benefits and harms of the HPV vaccines, we conducted a systematic review with meta-analyses of trial data from clinical study reports. | 12 |
| Impact of human papillomavirus vaccination on the clinical meaning of cervical screening results | Castle, Xie, Xue, et al., 2019 | Preventive Medicine | Cohort studies | One author is formerly recipient of grants on HPV vaccine research from Merck/SPMSD, and grants to his employer from Roche and Genomica. One author has received grants for his HPV vaccination studies from Merck & Co., Inc. and GSK Biologicals through his employers and Tampere University. | None stated | To describe the impact of HPV vaccination on risks of cervical precancer following a positive and negative screening among women who just started routine screening. | 8 |
| Death after quadrivalent human papillomavirus (HPV) vaccination: causal or coincidental? | Tomljenovic & Shaw, 2012 | Pharmaceutical Regulatory Affairs: Open Access | Non-experimental evaluations (case study) | None stated | None stated | To determine whether or not some serious autoimmune and neurological ADRs following HPV vaccination are causal or merely coincidental and to validate a biomarker-based immunohistochemical protocol for assessing causality in case of vaccination-suspected serious adverse neurological outcomes. | 6 |
| Critical analysis of reference studies on the toxicokinetics of aluminum-based adjuvants | Masson, Crépeaux, Authier, Exley, & Gherardi, 2018 | Journal of Inorganic Biochemistry | Animal models | None stated | None stated | This study used aluminum salts resembling those used in vaccines but ignored adjuvant uptake by cells that was not fully documented at the time. | 6 |
| Autonomic dysfunction and HPV immunization: an overview | Blitshteyn, Brinth, Hendrickson & Martinez-Lavin, 2018 | Immunologic Research | Scoping review, narrative reviews, rapid reviews | None stated | One author has served as a medical expert witness on cases of POTS and other neurologic syndromes after Gardasil vaccine. | To review the case series reported from several countries describing patients with suspected severe side effects to the HPV vaccines. | 4 |
| Pancreatitis after human papillomavirus vaccination: a matter of molecular mimicry | Bizjak, Bruck, Praprotnik, Dahan, Shoenfeld, 2017 | Immunologic Research | Non-experimental evaluations (case study) | None stated | One author has served as an expert witness in cases involving adverse vaccine reaction in the no-fault USA National Vaccine Injury Compensation Program | To describe the case of a 20-year-old man developed severe abdominal pain 1 week after being vaccinated with the first dose of quadrivalent human papillomavirus (HPV) vaccine (Gardasil). | 4 |
| Has the HPV vaccine approval ushered in an era of over-prevention? | Riva & Spinosa, 2020 | Journal of Scientific Practice and Integrity | Scoping review, narrative reviews, rapid reviews | None stated | Both authors have authored an investigative book about the HPV vaccines, a critical comment on Cochrane HPV vaccine, methodology, and a Letter to Editor on this topic in BMJ-EBM. | To investigate the impact of US regulators’ choices on the quality of available evidence regarding the vaccine’s efficacy in preventing high-grade cervical lesions, which are precursors of cervical cancer. | 4 |
| Behavioral abnormalities in female mice following administration of aluminum adjuvants and the human papillomavirus (HPV) vaccine Gardasil | Inbar et al., 2017 | Immunologic Research | Animal models | None stated | One author has acted as a consultant for the no-fault US National Vaccine Injury Compensation Program. Another author has served as an expert witness in cases involving adverse reactions following qHPV vaccine administration. | To evaluate the effects of Al adjuvant and the HPV vaccine Gardasil versus the true placebo on behavioral and inflammatory parameters in female mice | 4 |
| Demyelinating disease and polyvalent human papillomavirus vaccination | Chang, Campagnolo, Vollmer, Bomprezzi, 2011 | Journal of Neurology, Neurosurgery & Psychiatry | Non-experimental evaluations (case study) | None stated | None stated | To describe two cases whose initial presentation of CNS demyelination followed in close time relationship the administration of Gardasil vaccine and we discuss their possible association. | 4 |
| Demyelinating disease and vaccination of the human papillomavirus | Soria et al., 2011 | Nota Clinica | Non-experimental evaluations (case study) | Not available in English | Not available in English | To describe the cases of four young women that developed demyelinating disease after the vaccination of the HPV, with a rank of time between the administration of the dose and the development of the clinical of seven days to a month, with similar symptoms with the successive doses. | 4 |
| Shift in prevalence of HPV types in cervical cytology specimens in the era of HPV vaccination | Fischer et al., 2016 | Oncology Letters | Cohort studies | None stated | None stated | To evaluate the prevalence of important HPV types in a local cohort of participants from Southern Bavaria, and analyzed the HPV type distribution in participants with abnormal cytological diagnostic findings, who were separated by various age groups. | 3 |
