# Supplementary material for: The Role of Scientific Research in Human Papillomavirus Vaccine Discussions on Twitter: Social Network Analysis
Source: JMIR Infodemiology. 2024 May 9;4:e50551. doi: 10.2196/50551 (PMC11117132; doi:10.2196/50551)
Supplement: Multimedia Appendix 2 [file infodemiology_v4i1e50551_app2.docx]

Supplementary Material B: Summary of Top 20 Most Shared Scientific Articles on Twitter by Vaccine Confident Community

| Article Name | Authors and Year | Publication Name | Study Design | Conflict of Interest: Financial | Conflict of Interest: Personal | Research Objective | Shares on Twitter |
| --- | --- | --- | --- | --- | --- | --- | --- |
| Prevalence of cervical disease at age 20 after immunisation with bivalent HPV vaccine at age 12-13 in Scotland: retrospective population study | Palmer, et al., 2019 | British Medical Journal | Cohort studies | One author has received travel monies from both Merck and GSK to attend conferences. One author’s institution has received funding to deliver research from: Qiagen, Hologic, Selfscreen, GeneFirst, Euroimmun, Cepheid, Genomica, and LifeRiver. | None stated | To quantify the effect on cervical disease at age 20 years of immunisation with bivalent human papillomavirus (HPV) vaccine at age 12-13 years. | 382 |
| HPV vaccination and the risk of invasive cervical cancer | Lei, et al., 2020 | The New England Journal of Medicine | Cohort studies | One author has received a grant from Merck for HPV vaccine research. Another author’s employer has received research grants from a vaccine manufacturer, Sanofi Pasteur MSD SNC. Another author received a grant from Merck for HPV vaccine research. Another author received a grant from Merck for HPV vaccine research. | None stated | To assess the association between HPV vaccination and the risk of invasive cervical cancer, controlling for age at follow-up, calendar year, county of residence, and parental characteristics, including education, household income, mother’s country of birth, and maternal disease history. | 233 |
| Anxiety and distress following receipt of results from routine HPV primary testing in cervical screening: The psychological impact of primary screening (PIPS) study | McBride, et al., 2020 | International Journal of Cancer | Cross sectional survey | None stated | None stated | To examine short‐term anxiety and distress in women receiving different results following routine human papillomavirus (HPV) primary testing at cervical screening | 57 |
| The remarkable impact of bivalent HPV vaccine in Scotland | Brotherton, 2019 | British Medical Journal | Commentaries, expert opinions, editorials | The author’s employer has received partial, unrestricted support (in the form of equipment) to conduct a randomised trial of primary HPV screening from Roche Molecular Systems. | None stated | To describe the findings of a recently published article on the effectiveness of the HPV vaccine in preventing cervical cancer among young women in Scotland. | 45 |
| Human papilloma virus vaccine and primary ovarian failure: another facet of the autoimmune/inflammatory syndrome induced by adjuvants | Colafrancesco, Perricone, Tomljenovic, & Shoenfeld, 2013 | American Journal of Reproductive Immunology | Non-experimental evaluations (case study) | None stated | One of the authors has served as an expert witness in cases involving adverse vaccine reaction in the no-fault U.S. National Vaccine Injury Compensation Program. | To examine auto-immune/inflammatory syndrome reported in the cases of 3 young women who received an HPV vaccine. | 40 |
| HPV vaccine: high coverage could eradicate cervical cancer within decades, say researchers | Torjesen, 2019 | British Medical Journal | Commentaries, expert opinions, editorials | None stated | None stated | To describe the findings of a recently published meta-analysis on the impact of HPV vaccination programs for adolescent girls on precancerous cervical lesions and anogenital warts. | 35 |
| [RETRACTED] A lowered probability of pregnancy in females in the USA aged 25-29 who received a human papillomavirus vaccine injection | DeLong, 2018 | Journal of Toxicology and Environmental Health | Cohort studies | None stated | The author filed a claim under the Vaccine Injury Compensation Program on behalf of her daughter. The Special Master dismissed the claim due to untimely filing. The claim did not include the HPV vaccine. | To analyze information [birth outcomes] gathered in National Health and Nutrition Examination Survey, which represented 8 million 25-to-29-year-old women residing in the United States between 2007 and 2014 | 31 |
| Population-level impact and herd effects following the introduction of human papillomavirus vaccination programmes: updated systematic review and meta-analysis | Drolet, Bénard, Pérez, & Brisson, the HPV Vaccination Impact Study Group, 2019 | Lancet | Systematic Reviews and meta-analysis | None stated | None stated | To conduct a systematic review and meta-analysis of the population-level impact of vaccinating girls and women against human papillomavirus on HPV infections, anogenital wart diagnoses, and cervical intraepithelial neoplasia grade 2+ (CIN2+). | 21 |
| Primary cervical screening with high risk human papillomavirus testing: observational study | Matejka Rebolj, et al., 2019 | British Medical Journal | Cohort studies | One author attended meetings with various HPV assay manufacturers and received a fee for lecture from Hologic paid to employer. Another author received fees for lectures from Roche, Qiagen, and Hologic; conference registration, accommodation and travel from Sanofi Pasteur; consultancy fees and shareholder in Zilico; patent for electrical impedance spectroscopy in detection of cervical intraepithelial neoplasia with Zilico. The saem author also received fees for lectures from Beckton Dickinson and Roche and conference accommodation and travel from Hologic, Abbott, Becton Dickinson, and Roche. Another author received personal speaker bureau fees from Beckton Dickinson and personal medical advisory board fees from Zilico. Another author is employed by Norfolk and Norwich University Hospitals NHS Foundation Trust and received the speaker fees from Roche for conferences and travel and accommodation from Roche and Hologic for training and user group meetings. Another author received speaker fees from Roche, travel and accommodation from Roche for training, travel and accommodation from Abbott for a user group meeting, attended meetings with HPV assay manufacturers, and received kits for assay validation from Roche, Abbott, Hologic, Becton Dickinson, and Cepheid. | None stated | To provide the first report on the main outcomes from the prevalence and incidence rounds of a large pilot of routine primary high risk human papillomavirus (hrHPV) testing in England, compared with contemporaneous primary liquid based cytology screening. | 20 |
| Impact of HPV vaccine hesitancy on cervical cancer in Japan: a modelling study | Simms, Hanley, Smith, Keane, & Canfell, 2020 | The Lancet | Cohort studies | One of the authors is co-principal investigator of an unrelated investigator-initiated trial of cytology and primary human papillomavirus screening in Australia, which is conducted and funded by the VCS Foundation, a government-funded health promotion charity. In 2013, the VCS Foundation received equipment and a funding contribution for the Compass trial from Roche Molecular Systems and Ventana. | None stated | We aimed to quantify the impact of this vaccine hesitancy crisis, and the potential health gains if coverage can be restored. | 19 |
| Monitoring the safety of quadrivalent human papillomavirus vaccine: findings from the Vaccine Safety Datalink | Gee, et al., 2011 | Vaccine | Cohort studies | One author has received research support from GlaxoSmithKline,Merck & Co., Sanofi Pasteur, Wyeth (Pfizer), and Novartis; Another author has received funding from GlaxoSmithKline for a study of cervical procedures. Another author has worked on grants funded by Merck & Co., Wyeth (Pfizer), Novartis, Sanofi Pasteur, GlaxoSmithKline, and MedImmune (now AstraZeneca). | None stated | To detect associations between HPV4 exposure and pre-specified outcomes. | 17 |
| Long-term antibody response to human papillomavirus vaccines: Up to 12 years of follow-up in the Finnish Maternity Cohort | Artemchuk, et al., 2019 | The Journal of Infectious Diseases | Cohort studies | Three authors received grants for the HPV vaccination studies from Merck & Co, Inc and two authors received grants from GSK Biologicals. | None stated | To conduct an independent head-to-head comparison of Gardasil- and Cervarix-induced antibody levels up to 12 years post-vaccination | 16 |
| Variations in HPV function are associated with survival in squamous cell carcinoma | Gleber-Netto , et al., 2019 | JCI Insight | Cohort studies | None stated | None stated | To explore the association between HPV function and molecular and clinical phenotypes. | 16 |
| Eliminating HPV-caused cancers in Europe: Achieving the possible | Baker, Kelly, Medeiros, Morrissey, & Price, 2021 | Journal of Cancer Policy | Commentaries, expert opinions, editorials | None stated | None stated | To outline the prevention steps needed to meet the Member States goal of HPV vaccination and cervical cancer elimination in the EU. | 15 |
| Socio-demographic correlates of cervical cancer risk factor knowledge among screening non-participants in Great Britain | Ryan, Marlow, & Waller, 2019 | Preventive Medicine | Cross sectional survey | None stated | None stated | To identify knowledge gaps that could be targeted in screening information materials or public education campaigns. | 14 |
| Vaccine uptake and prevalence of HPV related cancers in US men | Kardas-Nelson, 2019 | British Medical Journal | Commentaries, expert opinions, editorials | None stated | None stated | To explore why men in the USA are not receiving the HPV vaccine despite it protecting against cancerous strains of the leading STI in the US. | 14 |
| Safety of quadrivalent human papillomavirus vaccine administered routinely to females | Klein, et al., 2012 | Archives of Pediatrics & Adolescent Medicine | Cohort studies | This study was funded by Merck & Co. One author received research support from Merck & Co, GlaxoSmithKline, Pfizer, Novartis, and sanofipasteur for unrelated studies. Two other authors received research funding from Merck & Co for another study related to HPV4. Another author served as an unpaid consultant to Merck & Co. Another author received research funding from Merck&Co, Amgen, and Pfizer for unrelated studies. Two authors are employees of Merck & Co. Role of the Sponsor: The study sponsor, Merck & Co, provided substantial input into the study design and analytic plan. | None stated | To assess the safety of the quadrivalent human papillomavirus vaccine (HPV4) in females following routine administration. | 12 |
| TCR-engineered T cells targeting E7 for patients with metastatic HPV-associated epithelial cancers | Nagarsheth, et al., 2021 | Nature Medicine | Quasi-experimental study | One author is an inventor on the NIH patent for the E7 TCR and other NIH patents in the field of immunotherapy. The same author also receives research funding through an NCI Collaborative Research and Development Agreement with Kite Pharma. | None stated | To conduct a first-in-human, phase 1 clinical trial of T cells engineered with a T cell receptor targeting HPV-16 E7 for the treatment of metastatic human papilloma virus-associated epithelial cancers | 12 |
| Cost‐effectiveness analysis of primary human papillomavirus testing in cervical cancer screening: Results from the HPV FOCAL Trial | Cromwell, et al., 2021 | Cancer Medicine | RCTs | None stated | None stated | To conduct a cost-effectiveness analysis based on the HPV FOCAL trial to estimate the incremental cost per detected high-grade cervical intraepithelial neoplasia of grade 2 or worse lesions (CIN2+). | 11 |
| State statutes and regulations related to human papillomavirus vaccination | Hoss, Meyerson, & Zimet, 2019 | Human Vaccines & Immunotherapeutics | Scoping review, narrative reviews, rapid reviews | Within the last year one author received an honorarium from Sanofi  Pasteur for work on the Adolescent Immunization Initiative and received  travel support from Merck to attend a conference on HPV vaccination. | None stated | To study of state HPV  vaccination requirements that derived data using legal epidemiological methods across statutes and regulations on  a breadth of topical areas including mandates, education,  scope of practice, and financing | 11 |
