# Supplementary figures and images for: The Role of Scientific Research in Human Papillomavirus Vaccine Discussions on Twitter: Social Network Analysis
Source: JMIR Infodemiology. 2024 May 9;4:e50551. doi: 10.2196/50551 (PMC11117132; doi:10.2196/50551)

Supplementary Material C: Retweet Network Map of HPV Immunization Conversations (n=596,987)


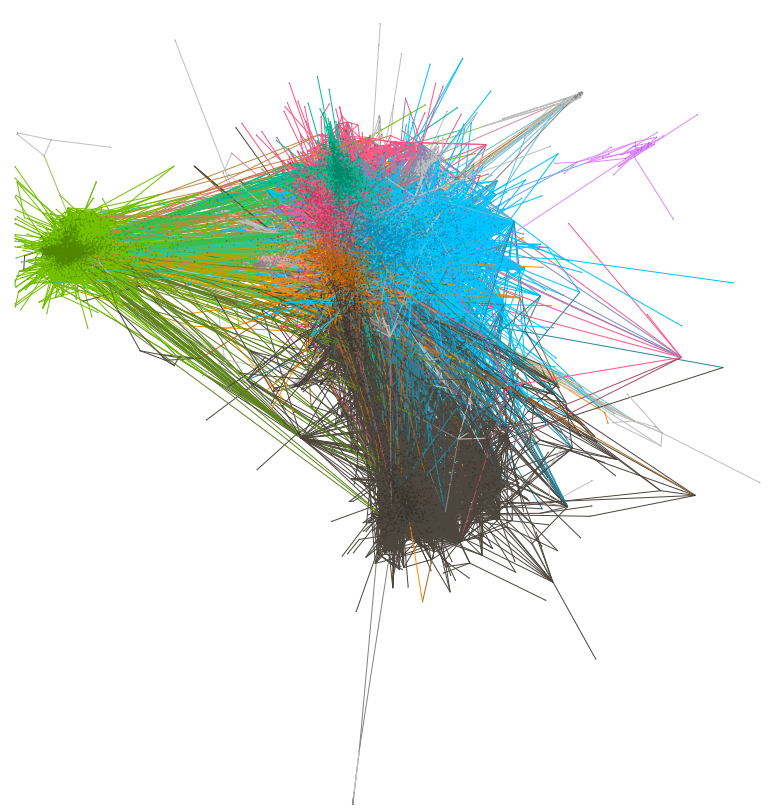

Supplement: Multimedia Appendix 3 [file infodemiology_v4i1e50551_app3.docx]
